# Acute and chronic stress differentially regulate cyclin-dependent kinase 5 in mouse brain: implications to glucocorticoid actions and major depression

**DOI:** 10.1038/tp.2015.72

**Published:** 2015-06-09

**Authors:** A Papadopoulou, T Siamatras, R Delgado-Morales, N D Amin, V Shukla, Y-L Zheng, H C Pant, O F X Almeida, T Kino

**Affiliations:** 1 Program in Reproductive and Adult Endocrinology, *Eunice Kennedy Shriver* National Institute of Child Health and Human Development, National Institutes of Health, Bethesda, MD, USA; 2 NeuroAdaptations Group, Max Planck Institute of Psychiatry, Munich, Germany; 3 Neuronal Cytoskeletal Protein Regulation Section, National Institute of Neurological Disorders and Stroke, Bethesda, MD, USA; 4 Division of Experimental Biology, Department of Experimental Therapeutics, Sidra Medical and Research Center, Doha, Qatar

## Abstract

Stress activates the hypothalamic–pituitary–adrenal axis, which in turn increases circulating glucocorticoid concentrations and stimulates the glucocorticoid receptor (GR). Chronically elevated glucocorticoids by repetitive exposure to stress are implicated in major depression and anxiety disorders. Cyclin-dependent kinase 5 (CDK5), a molecule essential for nervous system development, function and pathogenesis of neurodegenerative disorders, can modulate GR activity through phosphorylation. We examined potential contribution of CDK5 to stress response and pathophysiology of major depression. In mice, acute immobilized stress (AS) caused a biphasic effect on CDK5 activity, initially reducing but increasing afterwards in prefrontal cortex (PFC) and hippocampus (HIPPO), whereas chronic unpredictable stress (CS) strongly increased it in these brain areas, indicating that AS and CS differentially regulate this kinase activity in a brain region-specific fashion. GR phosphorylation contemporaneously followed the observed changes of CDK5 activity after AS, thus CDK5 may in part alter GR phosphorylation upon this stress. In the postmortem brains of subjects with major depression, CDK5 activity was elevated in Brodmann's area 25, but not in entire PFC and HIPPO. Messenger RNA expression of glucocorticoid-regulated/stress-related genes showed distinct expression profiles in several brain areas of these stressed mice or depressive subjects in which CDK5-mediated changes in GR phosphorylation may have some regulatory roles. Taken together, these results indicate that CDK5 is an integral component of stress response and major depression with regulatory means specific to different stressors, brain areas and diseases in part through changing phosphorylation of GR.

## Introduction

Unpredicted short- and long-term stressful events have a significant influence over our daily activities.^1, 2, 3^ The physiological response to stress includes integration of neural and humoral information at multiple levels in the brain and periphery, with the hypothalamic–pituitary–adrenal (HPA) axis playing a major role in coordination of central and peripheral components.^4^ Glucocorticoids secreted from the adrenal cortex in response to stress and following activation of the HPA axis, not only facilitate appropriate behavioral responses to stress, but also generate adaptive responses in intermediary metabolism, feeding, immunity and reproduction.^1, 5^ Stress-induced increase in glucocorticoid secretion is normally curtailed through a negative feedback mechanism functional in the HPA axis to maintain overall homeostasis.^2^ However, glucocorticoid secretion is sometimes unrestrained and maladaptive if the stress stimulus exceeds threshold of individual quality, intensity and/or chronicity, leading to development of an array of adverse effects, such as mood alteration, induction of anxiety and cognitive dysfunction, as well as immune suppression, osteoporosis and central obesity-associated insulin resistance and hyperlipidemia.^2, 5, 6^

The glucocorticoid receptor (GR), a member of the nuclear hormone receptor superfamily, mediates actions of circulating glucocorticoids in local tissues.^6, 7^ GR is expressed virtually in all organs and tissues including the central nervous system, and acts as a ligand-dependent transcription factor for these steroids.^8, 9^ Upon binding to ligand glucocorticoids, GR changes transcriptional rates of glucocorticoid-responsive genes positively and negatively by communicating with specific DNA sequences located in their promoter regions and/or with numerous transcriptional regulatory molecules, including other transcription factors, transcriptional cofactors and the RNA polymerase II.^6^ GR consists of three subdomains, N-terminal, middle DNA-binding and C-terminal ligand-binding domain.^9^ Transcriptional activity of GR is regulated through a number of posttranslational modifications, including phosphorylation, acetylation, SUMOylation and ubiquitylation, to adjust its diverse actions to needs of local organs and tissues.^6^

The cyclin-dependent kinase 5 (CDK5) is a serine/threonine kinase and a member of the cyclin-dependent kinase family.^10, 11^ Like other family members, CDK5 is activated through formation of a heterodimer with its partner molecules, such as p35 and p39.^12, 13^ CDK5 is essential for neuronal development and survival.^12, 13, 14, 15, 16, 17, 18^ In addition to these physiologic actions, aberrant CDK5 activation has a pivotal role in the pathogenesis of neurodegenerative disorders, such as Parkinson's disease, amyotrophic lateral sclerosis and Alzheimer's disease.^10^ In the latter condition, aberrantly activated CDK5 contributes to the hyperphosphorylation of the cytoskeletal protein tau and subsequent neurodegeneration.^19^ Activated CDK5 may also contribute to stress-induced depression-like behavior in rodents, in line with our previous suggestion that stress may trigger pathological processes shared by Alzheimer's disease and major depression.^20, 21, 22, 23^

We previously reported that CDK5 phosphorylates multiple serine residues of the human GR, such as serines 203, 211 and 213 (serines 212, 220 and 224 of mouse GR) located in its N-terminal domain, and modulates GR-induced transcriptional activity in rat primary neuronal cells.^7, 24^ We therefore examined the CDK5 activity and its protein levels in prefrontal cortex (PFC) and hippocampus (HIPPO) of the mice exposed to acute or chronic stress, as well as levels of phosphorylated GR and messenger RNA (mRNA) expression of 10 glucocorticoid-responsive and/or stress-related genes. We further examined these parameters in postmortem brains of the subjects with major depression. Our results suggest that acute and chronic stress differentially regulate CDK5 activity/expression in a brain area-specific fashion, which may further contribute to pathophysiology of stress-triggered major depression.

## Materials and methods

### Animal treatments and sampling of brain tissues

All animal procedures used in this manuscript were approved by the Government of Upper Bavaria, and were accordance with European Union Directive 2010/63/EU. Male mice of the C57Bl6/6NCrl strain, aged 12–14 weeks were maintained (three to four per cage) under standard conditions (21 °C; 12:12 light–dark cycle, lights on at 0600 h) at least for 2 weeks before the experiments. Mice were then randomly assigned to one of the following experimental groups: unstressed control (control, *n*=10 for each time point), acute stress (AS, *n*=8 for each time point), chronic unpredictable stress (CS, *n*=10 for each time point) or acute corticosterone-injected (CORT injection or CI, *n*=10 for each time point). The CI group received a single intraperitoneal injection of corticosterone (20 mg kg^−1^ in 40% cyclodextrin). Mice of the AS group were immobilized for 1 h in a 50 ml Falcon tube (with ventilation holes).^25^ Mice of the AS and CI groups, and their corresponding untreated controls were killed at 0, 1, 3 and 24 h after the treatment for obtaining blood and brain samples. The CS paradigm consisted of exposure to two different pairs of stressors either in the subjective day or in subjective night for 1 week, and then the same cycle was repeated for 4 weeks.^25, 26, 27^ The following seven pairs of stressors were applied during 1-week cycle: (Day 1) 1-h cage placement with orbital shaking (100 r.p.m.)+12-h damp bedding; (Day 2) 30-min immobilization in a 50 ml Falcon tube (as explained above for AS)+12-h placement in a tilted (45°) cage; (Day 3) 1-h exposure to white noise with overcrowding by placing four animals in a plastic box (10 × 10 × 5 cm) with ventilation holes+1-h cage placement with orbital shaking (100 r.p.m.); (Day 4) 1-h immobilization in a 50 ml Falcon tube+12-h placement in a tilted (45°) cage; (Day 5) 1-h exposure to white noise and overcrowding (see above)+12-h damp bedding (see above); (Day 6) 1-h cage placement with orbital shaking (100 r.p.m.)+12-h placement in a tilted (45°) cage; and (Day 7) 1-h immobilization in a 50 ml Falcon tube+24-h lights on (no dark period). Animals exposed to CS were killed on Day 29 by decapitation, which was carried out during the daily period of light, between 0800 and 1000 h. Trunk blood was collected at the time of euthanasia and serum corticosterone levels were measured in the harvested sera by using a commercial radioimmunoassay kit (MP Biomedicals, Costa Mesa, CA, USA). Brains were rapidly excised from the skull just after euthanasia, and PFC and HIPPO were dissected out, snap-frozen and stored at −80 °C until assays were performed (see below).

### Human brain samples

Thirty-three cryopreserved human brain samples of particular brain areas (13 HIPPO, eight PFC, four inferior frontal gyrus and eight Brodmann's area 25 (BA25)) obtained from eight deceased patients (five males and three females) with major depression and 14 control subjects (eight males and five females) without any psychiatric disorders but with other disorders, such as cancer, heart failure and chronic obstructive pulmonary disease, were donated from the Netherlands Brain Bank (Amsterdam, The Netherlands) and from the Human Brain and Spinal Fluid Resource Center of the West L.A. Healthcare Center (Los Angeles, CA, USA). Medication records were not available. The mean ages of disease and control subjects were 67.5±5.09 (s.e.) and 74.7±3.50, respectively (*P*=0.244, not significant).

### Brain sample preparation for CDK5 kinase assays and western blots

Cryopreserved brain samples were crushed in a mortar and homogenized by using a dounce homogenizer and the NE-PER Nuclear and Cytoplasmic Extraction Kit (PIERCE Biotechnology, Rockford, IL, USA). Protein concentrations were measured by using the BCA Protein Assay Kit (PIERCE Biotechnology). Protein extracts were stored at −80 °C until use.

### CDK5 kinase assay

CDK5 kinase assays were performed as previously described.^28^ Briefly, homogenized brain samples were incubated overnight at 4 °C with the anti-CDK5 antibody (C8; Santa Cruz Biotechnology, Santa Cruz, CA, USA), and the antibody-CDK5 complex was precipitated with the Protein A-Sepharose beads for 2 h at 4 °C. Immunoprecipitates were then washed three times with the lysis buffer, and then once with the kinase buffer containing 20 mM Tris-HCl (pH 7.4), 1 mM EDTA, 1 mM EGTA, 10 mM MgCl_2_, 10 mM sodium fluoride and 1 mM sodium orthovanadate. CDK5 kinase assays were performed in the same buffer containing 1 mM DTT, 0.1 mM ATP and 0.185 MBq of [γ-^32^P]ATP with 20 ng of histone H1 as a control substrate. The reaction was performed in a final volume of 50 μl, and was incubated for 60 min at 30 °C, stopped subsequently by addition of 10% SDS sample buffer followed by heating at 95 °C for 5 min. Twenty-five microliter aliquots of the reaction mixture were plotted on a Whatman p80 paper, washed, dried and were placed in a scintillation vial for counting.

### Western blot

Western blots were performed for evaluating expression levels of CDK5, total GR and the GR phosphorylated at serine 211 (human) or 220 (mouse). Western blots were also conducted for β-actin as an internal control. Proteins were separated on 4–12% NuPAGE Bis-Tris gels (Invitrogen, Carlsbad, CA, USA), and were transferred to a nitrocellulose membrane. After transfer, the membrane was incubated with the appropriate primary antibody obtained from Santa Cruz Biotechnology (anti-GR, CDK5 and β-actin antibody) or the anti-GR antibody specifically reacting to the GR phosphorylated at serine 211 (human) or 220 (mouse) (Cell Signaling Technology, Danvers, MA, USA), followed by incubation with the horseradish peroxidase-conjugated secondary antibody. The blotted proteins were then detected with the Amersham ECL Detection Reagents (GE Healthcare Bio-Science, Piscataway, NJ, USA). Blotted membranes were used repeatedly for different antibodies by stripping bound antibodies with the Restore western blot stripping buffer (Thermo Fisher Scientific, Waltham, MA, USA). The optical density of bands was measured using the Image J software (National Institutes of Health, Bethesda, MD, USA). The band density was first corrected with the background density obtained from proximity of the target band in X-ray films. The optical density of proteins of interest was further normalized against those for β-actin (also adjusted with the same procedure) to obtain relative expression levels. Relative phosphorylated GR levels were calculated by correcting band density of phosphorylated GR with that of total GR.

### SYBR Green real-time PCR analysis for mRNA expression

Total RNA was purified from frozen brain samples using the RNeasy Mini Kit (Qiagen Sciences, Germantown, MD, USA). Complementary DNA was synthesized using the TaqMan Reverse Transcription Reagents and oligo-dT as a primer (Applied Biosystems, Foster City, CA, USA). PCR was then performed in the 7500 Real-time PCR system (Applied Biosystems), as previously described.^29, 30^ Amplification was performed in a three-step cycle: 95 °C for 10 min, then 40 cycles of the reaction consisting of denaturing at 95 °C for 15 s and annealing/extension at 60 °C for 1 min, followed by a melting curve analysis. Ten target genes, which are reported to be glucocorticoid-responsive and/or regulated by stressful stimuli (glucocorticoid-responsive/stress-related genes) were the arginine vasopressin (*AVP*),^31^ brain-derived neurotrophic factor (*BDNF*),^32, 33^ corticotropin-releasing hormone (*CRH*),^31, 34^ FBJ murine osteosarcoma viral oncogene homolog (*cFos*),^35^ *FOSB/ΔFOSB*,^36^ 5-hydroxytryptamine (serotonin) receptor 1A (*HTR1A*),^37^ inhibitor of DNA-binding 3 (*ID3*),^38^ nischarin,^39^ nuclear speckle splicing regulatory protein 1 (*NSRP1*),^40^ protein phosphatase 1 regulatory subunit 10 (*PPP1R10*)^41^ and serum/glucocorticoid-regulated kinase 1 (*SGK1*).^42^ Primer pairs used in SYBR Green real-time PCR were designed so that the sequence spanning between a foward and a reverse primer contains at least one intron. Their sequences are listed in Supplementary Table 1. C_t_ values of the examined genes were normalized with those of the ribosomal protein large P0 (*RPLP0*) gene, and fold changes were obtained by using the comparative C_t_ method.^24^

### Statistical analysis

For all the experiments/measurements, sample sizes were chosen to achieve enough statistical power. Experiments/measurements were repeated at least three times. Randomization and blinding procedures were not used for animal studies. All the data are presented as means±s.e. in figures. Statistical analysis was performed by using the Student's *t*-test with two-tailed value and one-way analysis of variance with Bonferroni correction in the GraphPad PRIZM Version 5 (GraphPad Software, San Diego, CA, USA). The data variance was similar between the groups compared in each statistical analysis. Statistical significance was set at *P*<0.05.

## Results

### Acute and chronic stress differentially modulate CDK5 activity in mouse prefrontal cortex and hippocampus

To evaluate impact of acute and chronic stress on CDK5 kinase activity, we focused on two brain areas, the PFC and the HIPPO, because they are both major targets of stress,^1^ and are implicated in mood, emotion and cognitive functions.^43, 44^ Since brain has a strong compensating activity and duration of stress significantly influences direction and magnitude of response,^2^ we used two stress stimuli, the acute (AS) and chronic stress (CS): practically, we respectively used 1 h immobilization and chronic unpredictable stress. The latter consists of four cycles of 7-day stress treatment in which two different types of stressors were applied each day. Since these stressors stimulate the HPA axis and increase serum corticosterone levels,^2^ we also injected this steroid intraperitoneally to examine the effect of systemic glucocorticoid elevation on these brain regions. Indeed, 1 h immobilization AS significantly increased serum corticosterone concentrations after 1 h, which returned to the basal levels in 24 h (Supplementary Figure 1a). Corticosterone injection (20 mg kg^−1^) markedly increased serum levels of this steroid, which also returned to the basal levels after 24 h, although vehicle injection itself mildly increased corticosterone levels as well (Supplementary Figure 1b).

**Figure 1:**
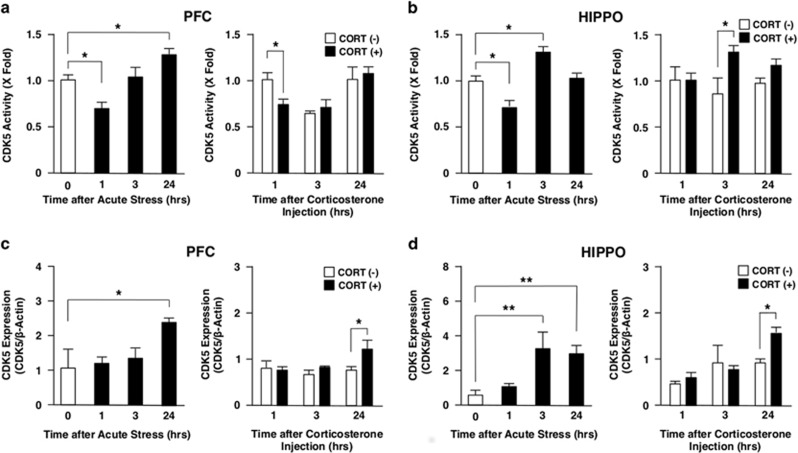
Acute stress and corticosterone injection differentially regulate CDK5 activity and protein expression in mouse PFC and HIPPO. Mice were immobilized in a 50 ml Falcon tube for 1 h or injected intraperitoneally with corticosterone (CORT, 20 mg kg^−1^) and were killed at 0, 1, 3 and 24 h after the treatment. CDK5 activity (**a** and **b**) and protein expression (**c** and **d**) were examined respectively with the CDK kinase assay and with the western blot using anti-CDK5 antibody. Bars represent mean±s.e. values of fold kinase activity against baseline (the condition at time 0 for AS, and the condition at 1 h in the absence of CORT for CORT injection) and the protein levels of CDK5. *P<0.05, **P<0.01, compared with the conditions indicated. HIPPO, hippocampus; PFC, prefrontal cortex.

Both in PFC and in HIPPO, AS developed a biphasic effect on the CDK5 activity: it suppressed the CDK5 activity 1 h after the exposure, while it reciprocally increased after 24 h in PFC (Figure 1a) and after 3 h in HIPPO (Figure 1b). CORT injection also suppressed the CDK5 activity 1 h after the injection in PFC (Figure 1a) and increased it after 3 h in HIPPO (Figure 1b), indicating a potential cause–effect relationship between elevated CORT secretion and the early changes in CDK5 activity observed upon exposure to AS.

We measured CDK5 protein levels using western blots to address the mechanisms underlying alteration of the CDK5 activity by AS. We found elevation of CDK5 protein levels 24 h and 3 h after exposure to AS in PFC and HIPPO, respectively. CDK5 protein levels mildly elevated only at a 24-h time point after CORT injection both in PFC and HIPPO (Figures 1c and d). These results indicate that alteration in the CDK5 protein levels may partly account for its elevated kinase activity observed at later time points upon AS, while the effects of AS on CDK5 protein expression were only partially mediated by secreted CORT.

**Figure 2:**
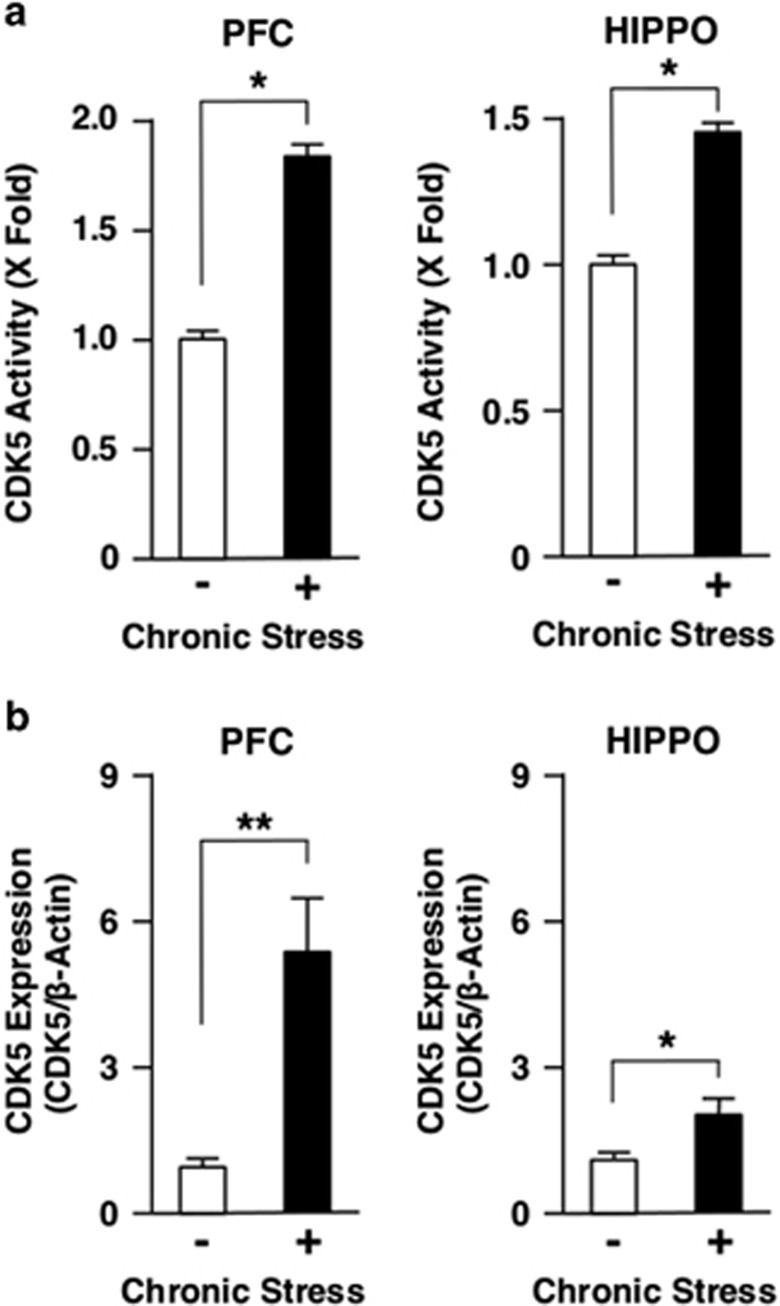
Chronic stress differentially regulates CDK5 activity and protein expression in mouse PFC and HIPPO. Mice were treated with chronic unpredictable stress for 28 days. CDK5 activity (**a**) and protein expression (**b**) were examined, respectively, with the CDK5 kinase assay and with the western blot using anti-CDK5 antibody. Bars represent mean±s.e. values of fold kinase activity against baseline (the condition in the absence of chronic stress) and the protein levels of CDK5 in the presence (closed bars) or absence (open bars) of chronic stress. *P<0.05, **P<0.01, compared with the conditions indicated. HIPPO, hippocampus; PFC, prefrontal cortex.

We next examined impact of CS to the CDK5 activity in mouse PFC and HIPPO. CS markedly increased the CDK5 activity both in these brain regions (Figure 2a), indicating that chronic exposure to stress is a strong stimulator of this kinase activity. CS also increased CDK5 protein levels both in PFC and HIPPO (Figure 2b).

### CDK5 may phosphorylate GR upon acute stress

**Figure 3:**
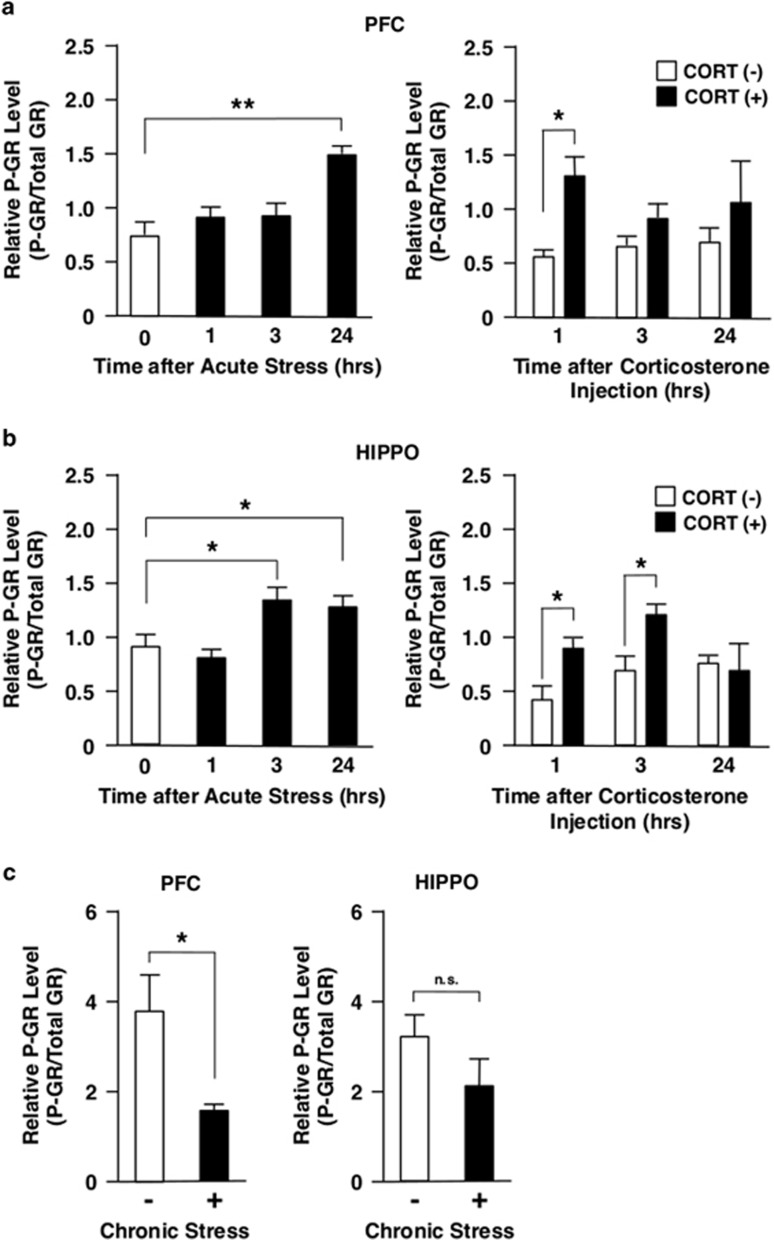
Acute stress, corticosterone injection and chronic stress differentially regulate phosphorylation of GR at serine 220 in mouse PFC and HIPPO. Mice were either immobilized in a 50 ml Falcon tube for 1 h (acute stress), injected with corticosterone (CORT, 20 mg kg^−1^) intraperitoneally (**a** and **b**) or treated with chronic unpredictable stress for 28 days (**c**) and their PFC and HIPPO were obtained at the time points indicated. The GR phosphorylated at serine 220 (P-GR) was examined with western blots using a specific antibody for this phosphorylated GR and relative P-GR levels were calculated by correcting band density of P-GR with that of total GR. Bars represent mean±s.e. values of relative P-GR levels. *P<0.05; **P<0.01; n.s., not significant, compared with the conditions indicated. GR, glucocorticoid receptor; HIPPO, hippocampus; PFC, prefrontal cortex.

We previously reported that CDK5 modulates GR-induced transcriptional activity through phosphorylation of this receptor.^24^ Thus, we examined the effect of acute or chronic stress on the phosphorylation of GR in PFC and HIPPO, using western blots with the antibody specifically reacting to the GR phosphorylated at serine 220, the residue known to be phosphorylated by CDK5.^24, 33^ AS increased GR phosphorylation 24 h after the exposure in PFC, while it suppressed GR phosphorylation after 1 h and increased after 3 h in HIPPO (Figures 3a and b). CORT injection increased GR phosphorylation after 1 h in PFC, while the injection increased it after 1 h and 3 h in HIPPO (Figures 3a and b), consistent with a well-known fact that GR is phosphorylated just after binding to ligand.^45^ These results indicate that the elevation of CDK5 activity by AS, respectively, observed after 24 h and 3 h in PFC and HIPPO may account for the elevation of GR phosphorylation in these areas. AS reduced CDK5 activity at 1 h time point in PFC and HIPPO, whereas CORT injection increased the phosphorylation at the same time point. Thus, mixture of these opposing activities observed upon AS might have caused the mild changes in the GR phosphorylation at early time points upon exposure to this stress. In contrast to AS, CS reduced GR phosphorylation in PFC but not in HIPPO (Figure 3c), while CDK5 activity was elevated in these brain areas (Figure 2b). Thus, unknown mechanisms appear to be functional in the regulation of GR phosphorylation following exposure to CS.

### Distinct mRNA expression profiles of the glucocorticoid-responsive/stress-related genes in acute and chronic stress

We examined mRNA expression profiles of a panel of glucocorticoid-responsive/stress-related genes in PFC and HIPPO upon exposure to AS, CS or CORT injection to examine whether there is a link between their expression and the observed alteration in the CDK5 kinase activity, its protein levels and phosphorylation of GR upon these stimuli. Indeed stimulation/suppression of transcript expression is a primary function of ligand-activated GR, while we showed in the current analysis that CDK5 in some part participated in the phosphorylation of GR upon AS.

**Figure 4:**
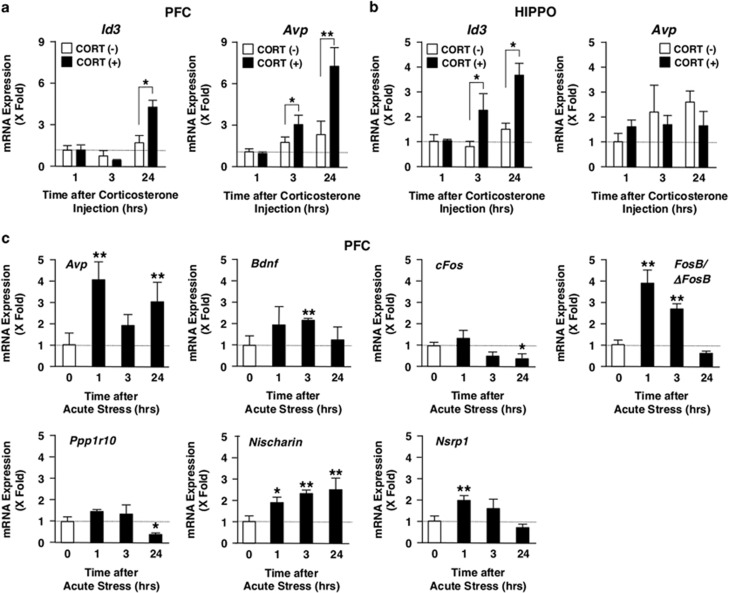

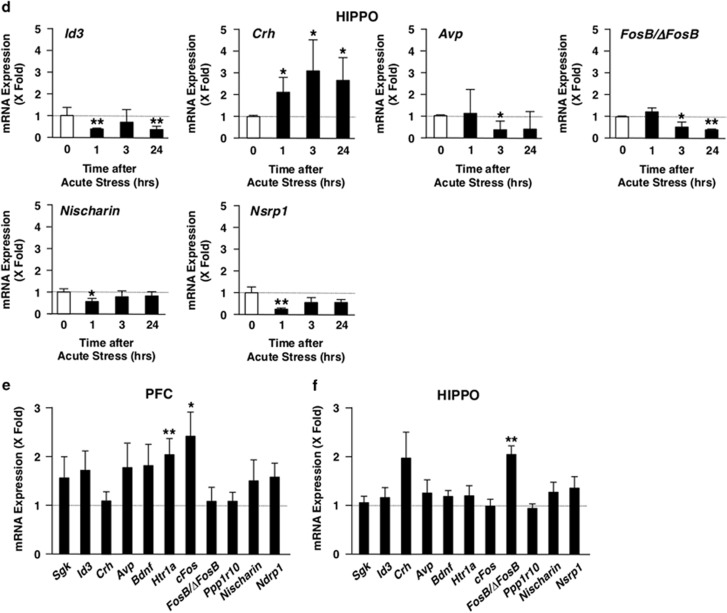
mRNA expression of glucocorticoid-responsive/stress-related genes in mice injected with corticosterone, or exposed to acute or chronic stress. (**a** and **b**) Corticosterone injection stimulates *Avp* and *Id3* mRNA expression, respectively, in mouse PFC and HIPPO. Mice were injected intraperitoneally with corticosterone (CORT, 20 mg kg^−1^), and were killed at 0, 1, 3 and 24 h after the treatment. mRNA expression of indicated genes was measured with the SYBR Green real-time PCR using their specific primers. Bars represent mean±s.e. values of fold mRNA expression of *Avp* and *Id3* in PFC (**a**) and HIPPO (**b**) in the presence (closed bars) or absence (open bars) of CORT injection. *P<0.05, **P<0.01, compared with the conditions indicated. (**c** and **d**) Acute stress differentially regulates mRNA expression of stress-related genes in mouse PFC and HIPPO. Mice were immobilized in a 50 ml falcon tube for 1 h, and were killed at 0, 1, 3 and 24 h after the treatment. mRNA expression of indicated genes was measured in PFC (**c**) and HIPPO (**d**) with the SYBR Green real-time PCR using their specific primers. Bars represent mean±s.e. values of fold mRNA expression of indicated genes in PFC (**c**) and HIPPO (**d**). *P<0.05, **P<0.01, compared with the conditions indicated. (**e** and **f**) Chronic stress differentially regulates mRNA expression of glucocorticoid-responsive/stress-related genes in mouse PFC and HIPPO. Mice were treated with chronic unpredictable stress for 28 days, and were killed after the treatment. mRNA expression of indicated genes was measured in PFC (**e**) and HIPPO (**f**) with SYBR Green real-time PCR using their specific primers. Bars represent mean±s.e. values of fold mRNA expression of indicated genes of stressed mice to that of control unstressed mice in PFC (**e**) and HIPPO (**f**). *P<0.05, **P<0.01, compared with the condition without chronic stress. HIPPO, hippocampus; mRNA, messenger RNA; PFC, prefrontal cortex.

Among the glucocorticoid-responsive/stress-related genes that we used, CORT injection increased in PFC *Id3* mRNA levels after 24 h and *Avp* mRNA levels after 3 and 24 h (Figure 4a), whereas it increased in HIPPO *Id3* mRNA abundance after 3 and 24 h (Figure 4b). These results suggest that *Id3*, and possibly *Avp* in PFC, is the gene responsive to the elevation of circulating corticosterone in these brain areas, although several other genes, such as *Bdnf*, *Crh* and *Sgk1*, are reported to respond to glucocorticoids *in vivo* or *in vitro*.^33, 34, 42, 46^ We then examined mRNA expression of all these glucocorticoid-responsive/stress-related genes in PFC and HIPPO of the mice exposed to AS or CS. Results of the genes demonstrating statistically significant changes after exposure to AS are shown in Figures 4c and d. We also displayed results of the all-examined genes in Supplementary Figures 2a and b for convenience of comparison. AS markedly increased mRNA expression of *Avp*, *Bdnf*, *FosB/ΔFosB*, *Nischarin* and *Rgd*, and suppressed *cFos* and *Ppp1r10* mRNA expression in PFC, whereas it increased *Crh* mRNA abundance and suppressed *Avp*, *Id3*, *FosB/ΔFosB* and *Nischarin* mRNA expression in HIPPO. These results suggest that the effect of AS on the mRNA expression of these genes is brain region-specific. Since the changes of mRNA expression profiles observed upon AS were completely different from those observed after CORT injection (particularly expression of *Id3* and *Avp*), some regulatory mechanisms in addition to the elevation of circulating corticosterone are functional in the former case. In contrast to the effect of AS and CORT injection, CS stimulated mRNA expression of *Htr1a* and *cFos* in PFC and that of *FosB/ΔFosB* in HIPPO (Figures 4e and f), indicating that CS has quite different regulatory activity on the mRNA expression of glucocorticoid-responsive/stress-related genes compared with AS.

### CDK5 activity was elevated in BA25 of depressive subjects

**Figure 5:**
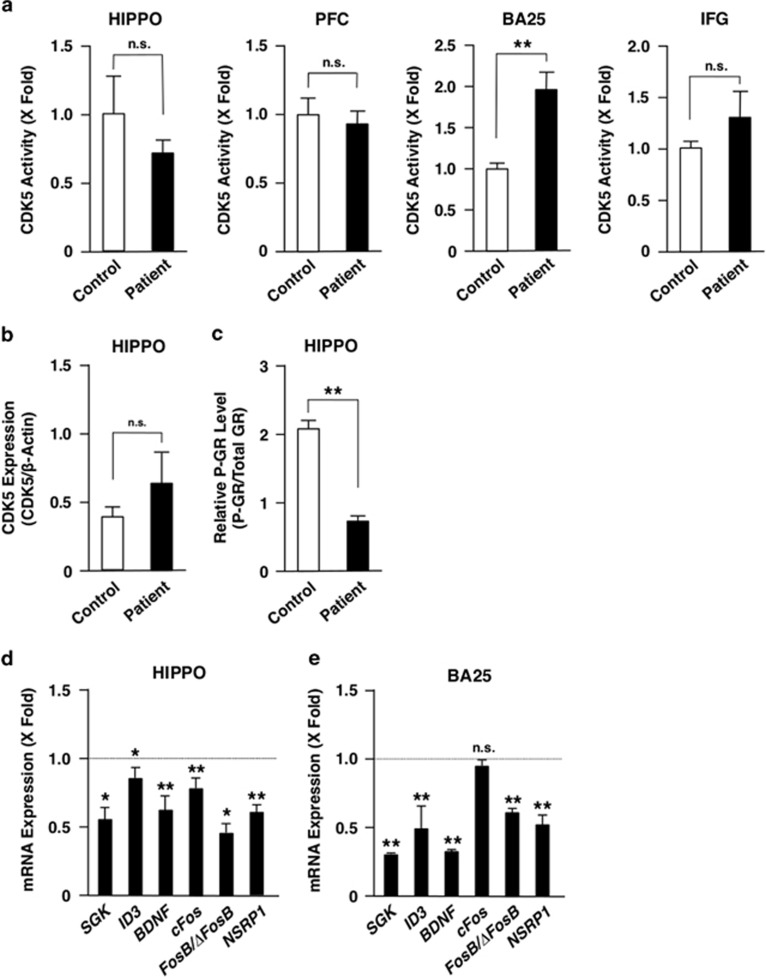
Depressive patients show variable CDK5 activity, its protein expression, GR phosphorylation and mRNA expression of glucocorticoid-responsive/stress-related genes in some brain areas. (**a**) Depressive patients demonstrate altered CDK5 acitivity in BA25, but not in PFC, HIPPO and IFG. The CDK5 acitivity was evaluated in HIPPO, PFC, BA25 and IFG of deceased patients with major depression or deceased control subjects. Bars represent mean±s.e. values of fold kinase activity in depressive patients (closed bars) or in control subjects (open bars; mean values of control subjects as ‘1'). **P<0.01, n.s., not significant, compared with the conditions indicated. (**b** and **c**) Depressive patients have unaltered CDK5 protein levels but reduced GR phosphorylation in HIPPO. Levels of the CDK5 protein (**b**) and the GR phosphorylated at serine 211 (P-GR) (**c**) were evaluated in HIPPO of deceased patients with major depression or deceased control subjects. Bars represent mean±s.e. values of relative CDK5 protein and P-GR levels in depressive patients (closed bars) or in control subjects (open bars). **P<0.01; n.s., not significant, compared with the conditions indicated. (**d** and **e**) Depressive patients demonstrate altered mRNA expression of glucocorticoid-responsive/stress-related genes in HIPPO and BA25. mRNA expression of the indicated genes was measured in HIPPO (**d**) and BA25 (**e**) of deceased patients with major depression and deceased control subjects with SYBR Green real-time PCR using their specific primers. Bars represent mean±s.e. values of fold mRNA expression of indicated genes of depressive patients to those of control subjects. *P<0.05, **P<0.01; n.s., not significant, compared with control subjects. GR, glucocorticoid receptor; HIPPO, hippocampus; IFG, inferior frontal gyrus; mRNA, messenger RNA; PFC, prefrontal cortex.

Since chronic stress is associated with pathophysiology of major depression and GR is a potential target of anti-depressant drugs,^22, 47, 48^ we measured CDK5 activity in PFC and HIPPO of postmortem brains of subjects with major depression (Figure 5a). In addition, we measured CDK5 activity in two specific subregions of the PFC implicated in mood and symptoms related to mood disorders, namely the subgenual anterior cingulate cortex (BA25) and the inferior frontal gyrus (corresponding to BA44, 45 and 47) to avoid a possibility of overlooking the phenomena specific to these brain areas.^49, 50, 51, 52^ We found that the CDK5 activity was elevated in BA25 of these depressive subjects, while the activity in their PFC, HIPPO and inferior frontal gyrus was similar to that of control subjects. Our ability to detect the change in BA25 most likely reflects its ‘small size' and ‘dilution effects' when whole PFC was analyzed.

Due to little availability of brain samples of these subjects, we were able to examine CDK5 protein levels and GR phosphorylation only in HIPPO. CDK5 protein levels were increased in depressive patients compared with control subjects, while GR phosphorylation decreased in this brain area (Figures 5b and c).

We further examined mRNA expression of some glucocorticoid-responsive/stress-related genes in HIPPO and BA25 of postmortem brain samples of depressive patients and control subjects. mRNA expression of most of the genes successfully examined was suppressed in HIPPO (*SGK*, *ID3*, *BDNF*, *cFOS*, *FOSB/ΔFOSB* and *RGD*) and in BA25 (*SGK*, *ID3*, *BDNF*, *FOSB/ΔFOSB* and *RGD*) of the patients compared with control subjects (Figures 5d and e).

## Discussion

Stress is a well-known trigger of major depression and has been more recently implicated in the pathogenesis of cognitive disorders, including Alzheimer's disease.^53^ These pathological conditions are believed to reflect exposure of the brain to high levels of circulating glucocorticoids.^2^ Since we previously reported that CDK5 regulates GR transcriptional activity by phosphorylating this receptor,^24^ we examined in the current study the potential role of CDK5 in stress response by focusing on mouse PFC and HIPPO. We also used several postmortem brain tissues including those of PFC and HIPPO from human subjects with major depression. Our present work indicates a potentially important role of CDK5 in the molecular mechanisms underlying stress response and major depression. They show that CDK5 activity is differentially regulated by stress in different brain areas, with duration of stress being a key variable.

We found that acute stress caused a biphasic change in CDK5 activity initially suppressing but increasing afterwards in FPC and HIPPO. The mechanism(s) underlying this characteristic change is(are) not known, but glucocorticoid secreted in response to acute stress may participate in this regulation as CORT injection caused in some part similar changes. Acute stress-induced biphasic change in CDK5 activity further indicates presence of yet unknown biological pathway(s) that regulate(s) this kinase activity in a compensative fashion: the pathway through changing the CDK5 protein levels may be one potential mechanism based on our results, while those through phosphorylation of CDK5 itself, such as by Wee1 and Myt1, as well as through alteration in the level of CDK5 activators, such as p25, p35 and p39,^13^ might also have roles.

We found that chronic stress increased the CDK5 kinase activity and its protein levels in PFC and HIPPO. These results support an idea that CDK5 is a component of adaptive response against chronic stress, consistent with previous reports obtained in microarray studies.^54, 55^ Our results may also provide mechanistic explanation to the known link between chronic stress and cognitive impairment, and further, Alzheimer's disease. Indeed, individuals in chronic stress are more likely to develop cognitive impairment than those without stress, and exposure to chronic stress is a potential risk factor for the development of Alzheimer's disease in later life.^53, 56, 57^ Mice under chronic mild stress significantly impairs spatial memory and increases tau phosphorylation in hippocampus, while CDK5 is essential for adult hippocampal neurogenesis required for memory consolidation.^56, 58^ Thus, our results are in line with the emerging hypothesis that major depression and Alzheimer's disease lie in a common trajectory and that the two diseases may share common mechanisms.^20, 21, 22, 23^

Since chronic stress is associated with major depression,^47, 48^ we examined the CDK5 kinase activity in PFC and HIPPO in postmortem brains of patients with major depression. This analysis failed to reveal any differences between depressed and control subjects in these brain areas, while increased CDK5 activity was found in BA25. The latter finding is important because BA25, a subregion of the PFC, is implicated in major depression as well as treatment response.^49, 50, 51^ However, comparing or extrapolating data obtained in the mouse study to those found in the human study suffers some limitations to be considered; First, these species sometimes respond differently to stressors.^59^ Second, sampling procedures were different between them. Last, we used male mice in the animal study but enrolled both male and female subjects in the human study. It should be noted that stress appears to exert different effects on male and female rodent behavior,^60^ and that men and women may differ in their vulnerability to depression and expression of depressive symptoms.^61^

We examined influence of stress-activated CDK5 on the glucocorticoid/GR actions by evaluating levels of the GR phosphorylated at serine 211 (human) or 220 (mouse), known targets of CDK5. In our animal experiments, CDK5 appeared to participate in the phosphorylation of GR upon acute stress, indicating that CDK5 functions as a kinase for phosphorylating GR not only in *in vitro* cellular conditions but also in *in vivo* animals.^24^ We, however, did not see a correlation between CDK5 activity and GR phosphorylation in chronic stress, suggesting that this condition is much more complicated than acute stress in terms of GR phosphorylation. There are several kinases and phosphatases whose activity is regulated by exposure to stress, whereas several other cyclin-dependent kinases, p38 MAPK, c-jun N-terminal kinases and AMK-activated protein kinase share the same GR phosphorylation sites with CDK5.^7^ Chronic psychosocial stress is known to alter phosphorylation of these GR sites in PFC and HIPPO.^62, 63, 64, 65^ Further, the regulatory component of protein phosphatase 1, a known phosphatase for GR, were upregulated in the raphe nucleus of patients with major depression.^66, 67^ It is thus possible that these kinases/phosphatases participate in the phosphorylation and/or dephosphorylation of GR under chronic stress.

Upon exposure to acute stress, genes, such as *Avp, Bdnf, FosB/ΔFosB, Id3, Nsrp1 and Ppp1r10*, demonstrated a biphasic change in their mRNA expression in PFC and/or HIPPO, while CDK5 activity and GR phosphorylation also demonstrated in response to acute stress a biphasic profile similar or mirrored to the mRNA expression of these genes. This coincidence may suggest that CDK5 influenced in some degree their mRNA expression through phosphorylation of GR. Alternatively, CDK5/GR phosphorylation may share upstream regulatory pathway(s) with these genes.

In the mice under chronic stress, *Htr1a* and *cFos* mRNA were highly induced in PFC, while *FosB/ΔFosB* mRNA was elevated in HIPPO; a distinct set of genes compared with those induced by acute stress or CORT injection. Moreover, mRNA expression of most of the genes examined in human subjects was suppressed in HIPPO and BA25 of depressive subjects. These results thus indicate that acute, chronic stress and depression are quite different conditions in regard to mRNA expression of glucocorticoid-responsive and/or stress-related genes, and suggest presence of gene-regulatory mechanisms unique to each of these stimuli/disease, consistent with previous reports using the microarray analysis.^68, 69, 70^

## References

[bib1] Pego JM Sousa JC Almeida OF Sousa N Stress and the neuroendocrinology of anxiety disorders Curr Top Behav Neurosci 2010 2 97 117 21309108 10.1007/7854_2009_13

[bib2] Chrousos GP Stress and disorders of the stress system Nat Rev Endocrinol 2009 5 374 381 19488073 10.1038/nrendo.2009.106

[bib3] Nader N Chrousos GP Kino T Circadian rhythm transcription factor CLOCK regulates the transcriptional activity of the glucocorticoid receptor by acetylating its hinge region lysine cluster: potential physiological implications FASEB J 2009 23 1572 1583 19141540 10.1096/fj.08-117697 PMC2669420

[bib4] Chrousos GP The hypothalamic-pituitary-adrenal axis and immune-mediated inflammation N Engl J Med 1995 332 1351 1362 7715646 10.1056/NEJM199505183322008

[bib5] Chrousos GP Kino T Glucocorticoid action networks and complex psychiatric and/or somatic disorders Stress 2007 10 213 219 17514590 10.1080/10253890701292119

[bib6] Kino T De Martino MU Charmandari E Mirani M Chrousos GP Tissue glucocorticoid resistance/hypersensitivity syndromes J Steroid Biochem Mol Biol 2003 85 457 467 12943736 10.1016/s0960-0760(03)00218-8

[bib7] Kino T Tissue glucocorticoid sensitivity: beyond stochastic regulation on the diverse actions of glucocorticoids Horm Metab Res 2007 39 420 424 17578758 10.1055/s-2007-980193

[bib8] Burris TP The nuclear receptor superfamily Burris TP McCabe ERB(eds). Nuclear Receptors and Genetic Disease Academic Press: London, UK 2001 1 58

[bib9] Chrousos GP Kino T Intracellular glucocorticoid signaling: a formerly simple system turns stochastic Sci STKE 2005 2005 pe48 16204701 10.1126/stke.3042005pe48

[bib10] Su SC Tsai LH Cyclin-dependent kinases in brain development and disease Annu Rev Cell Dev Biol 2011 27 465 491 21740229 10.1146/annurev-cellbio-092910-154023

[bib11] Kesavapany S Li BS Amin N Zheng YL Grant P Pant HC Neuronal cyclin-dependent kinase 5: role in nervous system function and its specific inhibition by the Cdk5 inhibitory peptide Biochim Biophys Acta 2004 1697 143 153 15023357 10.1016/j.bbapap.2003.11.020

[bib12] Dhavan R Tsai LH A decade of CDK5 Nat Rev Mol Cell Biol 2001 2 749 759 11584302 10.1038/35096019

[bib13] Cheung ZH Ip NY Cdk5: a multifaceted kinase in neurodegenerative diseases Trends Cell Biol 2012 22 169 175 22189166 10.1016/j.tcb.2011.11.003

[bib14] Lai KO Ip NY Recent advances in understanding the roles of Cdk5 in synaptic plasticity Biochim Biophys Acta 2009 1792 741 745 19442718 10.1016/j.bbadis.2009.05.001

[bib15] Ledda F Paratcha G Ibanez CF Target-derived GFRα1 as an attractive guidance signal for developing sensory and sympathetic axons via activation of Cdk5 Neuron 2002 36 387 401 12408843 10.1016/s0896-6273(02)01002-4

[bib16] Paglini G Peris L Diez-Guerra J Quiroga S Caceres A The Cdk5-p35 kinase associates with the Golgi apparatus and regulates membrane traffic EMBO Rep 2001 2 1139 1144 11743029 10.1093/embo-reports/kve250 PMC1084168

[bib17] Smith DS Tsai LH Cdk5 behind the wheel: a role in trafficking and transport Trends Cell Biol 2002 12 28 36 11854007 10.1016/s0962-8924(01)02181-x

[bib18] Kwon YT Gupta A Zhou Y Nikolic M Tsai LH Regulation of N-cadherin-mediated adhesion by the p35-Cdk5 kinase Curr Biol 2000 10 363 372 10753743 10.1016/s0960-9822(00)00411-5

[bib19] Mandelkow EM Mandelkow E Biochemistry and cell biology of tau protein in neurofibrillary degeneration Cold Spring Harb Perspect Med 2012 2 a006247 22762014 10.1101/cshperspect.a006247 PMC3385935

[bib20] Zhong P Liu X Zhang Z Hu Y Liu SJ Lezama-Ruiz M Cyclin-dependent kinase 5 in the ventral tegmental area regulates depression-related behaviors J Neurosci 2014 34 6352 6366 24790206 10.1523/JNEUROSCI.3673-13.2014 PMC4004818

[bib21] Yu S Holsboer F Almeida OF Neuronal actions of glucocorticoids: focus on depression J Steroid Biochem Mol Biol 2008 108 300 309 17933520 10.1016/j.jsbmb.2007.09.014

[bib22] Anacker C Zunszain PA Cattaneo A Carvalho LA Garabedian MJ Thuret S Antidepressants increase human hippocampal neurogenesis by activating the glucocorticoid receptor Mol Psychiatry 2011 16 738 750 21483429 10.1038/mp.2011.26 PMC3121947

[bib23] Sotiropoulos I Catania C Riedemann T Fry JP Breen KC Michaelidis TM Glucocorticoids trigger Alzheimer disease-like pathobiochemistry in rat neuronal cells expressing human tau J Neurochem 2008 107 385 397 18691381 10.1111/j.1471-4159.2008.05613.x

[bib24] Kino T Ichijo T Amin ND Kesavapany S Wang Y Kim N Cyclin-dependent kinase 5 differentially regulates the transcriptional activity of the glucocorticoid receptor through phosphorylation: clinical implications for the nervous system response to glucocorticoids and stress Mol Endocrinol 2007 21 1552 1568 17440046 10.1210/me.2006-0345

[bib25] Patchev AV Fischer D Wolf SS Herkenham M Gotz F Gehin M Insidious adrenocortical insufficiency underlies neuroendocrine dysregulation in TIF-2 deficient mice FASEB J 2007 21 231 238 17135362 10.1096/fj.06-6952com

[bib26] Willner P Chronic mild stress (CMS) revisited: consistency and behavioural-neurobiological concordance in the effects of CMS Neuropsychobiology 2005 52 90 110 16037678 10.1159/000087097

[bib27] Bessa JM Ferreira D Melo I Marques F Cerqueira JJ Palha JA The mood-improving actions of antidepressants do not depend on neurogenesis but are associated with neuronal remodeling Mol Psychiatry 2009 14 764 773739. 18982002 10.1038/mp.2008.119

[bib28] Zheng YL Kesavapany S Gravell M Hamilton RS Schubert M Amin N A Cdk5 inhibitory peptide reduces tau hyperphosphorylation and apoptosis in neurons EMBO J 2005 24 209 220 15592431 10.1038/sj.emboj.7600441 PMC544899

[bib29] Kino T Tiulpakov A Ichijo T Chheng L Kozasa T Chrousos GP G protein β interacts with the glucocorticoid receptor and suppresses its transcriptional activity in the nucleus J Cell Biol 2005 169 885 896 15955845 10.1083/jcb.200409150 PMC2171637

[bib30] Ng SS Chang TH Tailor P Ozato K Kino T Virus-induced differential expression of nuclear receptors and coregulators in dendritic cells: implication to interferon production FEBS Lett 2011 585 1331 1337 21492741 10.1016/j.febslet.2011.04.001 PMC3101080

[bib31] Fuchsl AM Langgartner D Reber SO Mechanisms underlying the increased plasma ACTH levels in chronic psychosocially stressed male mice PLoS One 2013 8 e84161 24376791 10.1371/journal.pone.0084161 PMC3871658

[bib32] Ortiz JB Mathewson CM Hoffman AN Hanavan PD Terwilliger EF Conrad CD Hippocampal brain-derived neurotrophic factor mediates recovery from chronic stress-induced spatial reference memory deficits Eur J Neurosci 2014 40 3351 3362 25156382 10.1111/ejn.12703

[bib33] Kino T Jaffe H Amin ND Chakrabarti M Zheng YL Chrousos GP Cyclin-dependent kinase 5 modulates the transcriptional activity of the mineralocorticoid receptor and regulates expression of brain-derived neurotrophic factor Mol Endocrinol 2010 24 941 952 20357208 10.1210/me.2009-0395 PMC2870940

[bib34] Sharma D Bhave S Gregg E Uht R Dexamethasone induces a putative repressor complex and chromatin modifications in the CRH promoter Mol Endocrinol 2013 27 1142 1152 23671328 10.1210/me.2013-1079 PMC3706841

[bib35] Mifsud KR Gutierrez-Mecinas M Trollope AF Collins A Saunderson EA Reul JM Epigenetic mechanisms in stress and adaptation Brain Behav Immun 2011 25 1305 1315 21704151 10.1016/j.bbi.2011.06.005

[bib36] Garcia-Perez D Laorden ML Milanes MV Nunez C Glucocorticoids regulation of FosB/ΔFosB expression induced by chronic opiate exposure in the brain stress system PLoS One 2012 7 e50264 23185589 10.1371/journal.pone.0050264 PMC3503985

[bib37] Boyarskikh UA Bondar NP Filipenko ML Kudryavtseva NN Downregulation of serotonergic gene expression in the Raphe nuclei of the midbrain under chronic social defeat stress in male mice Mol Neurobiol 2013 48 13 21 23392607 10.1007/s12035-013-8413-y

[bib38] Konishi H Ogawa T Nakagomi S Inoue K Tohyama M Kiyama H Id1, Id2 and Id3 are induced in rat melanotrophs of the pituitary gland by dopamine suppression under continuous stress Neuroscience 2010 169 1527 1534 20600660 10.1016/j.neuroscience.2010.06.030

[bib39] Head GA Mayorov DN Imidazoline receptors, novel agents and therapeutic potential Cardiovasc Hematol Agents Med Chem 2006 4 17 32 16529547 10.2174/187152506775268758

[bib40] Olah J Vincze O Virok D Simon D Bozso Z Tokesi N Interactions of pathological hallmark proteins: tubulin polymerization promoting protein/p25, β-amyloid, and α-synuclein J Biol Chem 2011 286 34088 34100 21832049 10.1074/jbc.M111.243907 PMC3190826

[bib41] Charmandari E Chrousos GP Lambrou GI Pavlaki A Koide H Ng SS Peripheral CLOCK regulates target-tissue glucocorticoid receptor transcriptional activity in a circadian fashion in man PLoS One 2011 6 e25612 21980503 10.1371/journal.pone.0025612 PMC3182238

[bib42] Anacker C Cattaneo A Musaelyan K Zunszain PA Horowitz M Molteni R Role for the kinase SGK1 in stress, depression, and glucocorticoid effects on hippocampal neurogenesis Proc Natl Acad Sci USA 2013 110 8708 8713 23650397 10.1073/pnas.1300886110 PMC3666742

[bib43] McEwen BS Morrison JH The brain on stress: vulnerability and plasticity of the prefrontal cortex over the life course Neuron 2013 79 16 29 23849196 10.1016/j.neuron.2013.06.028 PMC3753223

[bib44] Preston AR Eichenbaum H Interplay of hippocampus and prefrontal cortex in memory Curr Biol 2013 23 R764 R773 24028960 10.1016/j.cub.2013.05.041 PMC3789138

[bib45] Orti E Bodwell JE Munck A Phosphorylation of steroid hormone receptors Endocr Rev 1992 13 105 128 1555532 10.1210/edrv-13-1-105

[bib46] Bennett MR Lagopoulos J Stress and trauma: BDNF control of dendritic-spine formation and regression Prog Neurobiol 2014 112 80 99 24211850 10.1016/j.pneurobio.2013.10.005

[bib47] Gold PW Goodwin FK Chrousos GP Clinical and biochemical manifestations of depression. Relation to the neurobiology of stress (1) N Engl J Med 1988 319 348 353 3292920 10.1056/NEJM198808113190606

[bib48] Gold PW Goodwin FK Chrousos GP Clinical and biochemical manifestations of depression. Relation to the neurobiology of stress (2) N Engl J Med 1988 319 413 420 3041279 10.1056/NEJM198808183190706

[bib49] Hamani C Mayberg H Stone S Laxton A Haber S Lozano AM The subcallosal cingulate gyrus in the context of major depression Biol Psychiatry 2011 69 301 308 21145043 10.1016/j.biopsych.2010.09.034

[bib50] Hamon M Blier P Monoamine neurocircuitry in depression and strategies for new treatments Prog Neuropsychopharmacol Biol Psychiatry 2013 45 54 63 23602950 10.1016/j.pnpbp.2013.04.009

[bib51] Pierrot-Deseilligny C From the abducens nucleus to spatial memory: an ocular motor journey Rev Neurol (Paris) 2005 161 549 565 16106806 10.1016/s0035-3787(05)85089-1

[bib52] Price JL Drevets WC Neural circuits underlying the pathophysiology of mood disorders Trends Cogn Sci 2012 16 61 71 22197477 10.1016/j.tics.2011.12.011

[bib53] Morimoto RI Stress, aging, and neurodegenerative disease N Engl J Med 2006 355 2254 2255 17124027 10.1056/NEJMcibr065573

[bib54] Liu Y Yang N Zuo P cDNA microarray analysis of gene expression in the cerebral cortex and hippocampus of BALB/c mice subjected to chronic mild stress Cell Mol Neurobiol 2010 30 1035 1047 20532976 10.1007/s10571-010-9534-8 PMC11498782

[bib55] Lee JH Ko E Kim YE Min JY Liu J Kim Y Gene expression profile analysis of genes in rat hippocampus from antidepressant treated rats using DNA microarray BMC Neurosci 2010 11 152 21118505 10.1186/1471-2202-11-152 PMC3009642

[bib56] Cuadrado-Tejedor M Ricobaraza A Del Rio J Frechilla D Franco R Perez-Mediavilla A Chronic mild stress in mice promotes cognitive impairment and CDK5-dependent tau hyperphosphorylation Behav Brain Res 2011 220 338 343 21238494 10.1016/j.bbr.2011.01.005

[bib57] Nihonmatsu-Kikuchi N Hayashi Y Yu XJ Tatebayashi Y Depression and Alzheimer's disease: novel postmortem brain studies reveal a possible common mechanism J Alzheimers Dis 2013 37 611 621 23948909 10.3233/JAD-130752

[bib58] Lagace DC Benavides DR Kansy JW Mapelli M Greengard P Bibb JA Cdk5 is essential for adult hippocampal neurogenesis Proc Natl Acad Sci USA 2008 105 18567 18571 19017796 10.1073/pnas.0810137105 PMC2587597

[bib59] Fernandez-Guasti A Fiedler JL Herrera L Handa RJ Sex, stress, and mood disorders: at the intersection of adrenal and gonadal hormones Horm Metab Res 2012 44 607 618 22581646 10.1055/s-0032-1312592 PMC3584173

[bib60] Dalla C Pitychoutis PM Kokras N Papadopoulou-Daifoti Z Sex differences in response to stress and expression of depressive-like behaviours in the rat Curr Top Behav Neurosci 2011 8 97 118 21769725 10.1007/7854_2010_94

[bib61] Bangasser DA Valentino RJ Sex differences in stress-related psychiatric disorders: neurobiological perspectives Front Neuroendocrinol 2014 35 303 319 24726661 10.1016/j.yfrne.2014.03.008 PMC4087049

[bib62] Mitic M Simic I Djordjevic J Radojcic MB Adzic M Gender-specific effects of fluoxetine on hippocampal glucocorticoid receptor phosphorylation and behavior in chronically stressed rats Neuropharmacology 2013 70 100 111 23353902 10.1016/j.neuropharm.2012.12.012

[bib63] Coffey ET Nuclear and cytosolic JNK signalling in neurons Nat Rev Neurosci 2014 15 285 299 24739785 10.1038/nrn3729

[bib64] Mehan S Meena H Sharma D Sankhla R JNK: a stress-activated protein kinase therapeutic strategies and involvement in Alzheimer's and various neurodegenerative abnormalities J Mol Neurosci 2011 43 376 390 20878262 10.1007/s12031-010-9454-6

[bib65] Sodero AO Trovo L Iannilli F Van Veldhoven P Dotti CG Martin MG Regulation of tyrosine kinase B activity by the cyp46/cholesterol loss pathway in mature hippocampal neurons: relevance for neuronal survival under stress and in aging J Neurochem 2011 116 747 755 21214568 10.1111/j.1471-4159.2010.07079.x

[bib66] Kerman IA Bernard R Bunney WE Jones EG Schatzberg AF Myers RM Evidence for transcriptional factor dysregulation in the dorsal raphe nucleus of patients with major depressive disorder Front Neurosci 2012 6 135 23087602 10.3389/fnins.2012.00135 PMC3475304

[bib67] DeFranco DB Qi M Borror KC Garabedian MJ Brautigan DL Protein phosphatase types 1 and/or 2A regulate nucleocytoplasmic shuttling of glucocorticoid receptors Mol Endocrinol 1991 5 1215 1228 1663212 10.1210/mend-5-9-1215

[bib68] Andrus BM Blizinsky K Vedell PT Dennis K Shukla PK Schaffer DJ Gene expression patterns in the hippocampus and amygdala of endogenous depression and chronic stress models Mol Psychiatry 2012 17 49 61 21079605 10.1038/mp.2010.119 PMC3117129

[bib69] Kang HJ Adams DH Simen A Simen BB Rajkowska G Stockmeier CA Gene expression profiling in postmortem prefrontal cortex of major depressive disorder J Neurosci 2007 27 13329 13340 18045927 10.1523/JNEUROSCI.4083-07.2007 PMC3763487

[bib70] Mehta D Menke A Binder EB Gene expression studies in major depression Curr Psychiatry Rep 2010 12 135 144 20425299 10.1007/s11920-010-0100-3 PMC2847693

