# Supplementary material for: Acute and chronic stress differentially regulate cyclin-dependent kinase 5 in mouse brain: implications to glucocorticoid actions and major depression
Source: Transl Psychiatry. 2015 Jun 9;5(6):e578–. doi: 10.1038/tp.2015.72 (PMC4490283; doi:10.1038/tp.2015.72)
Supplement: Supplementary Information [file tp201572x1.doc]

**Supplemental Materials**

**Acute and Chronic Stress Differentially Regulate Cyclin-dependent Kinase 5 in Mouse Brain: Implications to Glucocorticoid Actions and Major Depression**

Anna Papadopoulou^1^*, Thomas Siamatras^1^*, Raul Delgado-Morales^2^*, Niranjana D. Amin^3^, Varsha Shukla^3^, Ya-Li Zheng^3^, Harish C. Pant^3^, Osborne F.X. Almeida^2^ and Tomoshige Kino^1,4^

1: Program in Reproductive and Adult Endocrinology, *Eunice Kennedy Shriver* National Institute of Child Health and Human Development, National Institutes of Health, Bethesda, MD 20892, USA, 2: NeuroAdaptations Group, Max Planck Institute of Psychiatry, Munich 80804, Germany, 3: Neuronal Cytoskeletal Protein Regulation Section, National Institute of Neurological Disorders and Stroke, Bethesda, MD 20892, USA, 4: Experimental Biology, Sidra Medical and Research Center, Doha, Qatar

*: These authors contributed equally to this manuscript.

**CONTENTS**

**Supplemental Table 1.** Primer pairs used in SYBR Green PCR analysis.

**Supplemental Figure 1.** Changes of the serum corticosterone levels after acute stress or corticosterone injection in mice.

**Supplemental Figure 2.** Acute stress differentially regulates mRNA expression of stress-related genes in mouse PFC and HIPPO.

**Supplemental Table 1. Primer pairs used in SYBR Green PCR analysis.**

| **Gene name** |  | **Primer sequence** |
| --- | --- | --- |
| **Mouse** |  |  |
| *Avp* | Forward | 5’-CTGCCAGTCTGGCCAGAAG-3’ |
|  | Reverse | 5’-GGTGAGGCGGAAAAAACC-3’ |
| *Bdnf* | Forward | 5’-GAGTCTCCAGGACAGCAAAGC-3’ |
|  | Reverse | 5’-CTTGTCCGTGGACGTTTGC-3’ |
| *cFos* | Forward | 5’-GCTATATCCATGTACTGTAG-3’ |
|  | Reverse | 5’-GAAAACTGTTAATGTCAGAAC -3’ |
| *Crh* | Forward | 5’-GTACCTCGCAGAACAACAG-3’ |
|  | Reverse | 5’-GCAGACAGGGCGACAGAG-3’ |
| *FosB/ΔFosB* | Forward | 5’-AGGCAGAGCTGGAGTCGGAGAT-3’ |
|  | Reverse | 5’-GCCGAGGACTTGAACTTCACTCG-3’ |
| *Htr1a* | Forward | 5’-TACCAGGTGCTCAACAAGTG-3’ |
|  | Reverse | 5’-CTATAGGGTCGGTGATAGC-3’ |
| *Id3* | Forward | 5’-CTGCAGCGTGTCATAGAC-3’ |
|  | Reverse | 5’-*CAGATGAGCCTGGTCTAGC*-3’ |
| *Nsrp1* | Forward | 5’-GAGGAAACGGTGATGTCAG-3’ |
|  | Reverse | 5’-GTTCAAGGGTCCCAATG-3’ |
| *Nischarin* | Forward | 5’-GATGTCAAGTCCAAACTG-3’ |
|  | Reverse | 5’-CTTGGTTGGAGAGGATCC-3’ |
| *Ppp1r10* | Forward | 5’-GTGATGGCACGTTCACATCTG-3’ |
|  | Reverse | 5’-GTTAATGAAAGCTGGTCTCAC-3’ |
| *Sgk1* | Forward | 5’-GTTCTTGTAGCAAGGCAC-3’ |
|  | Reverse | 5’-CAGGAAAGGGTGCTTCAC-3’ |
| *Rplp0* | Forward | 5’-GAGGACCTCACTGAGATTCG-3’ |
|  | Reverse | 5’-CTGGAAGAAGGAGGTCTTCTC-3’ |
| **Human** |  |  |
| *BDNF* | Forward | 5’-GAGAAGAGTGATGACCATC-3’ |
|  | Reverse | 5’-GTCCTCGGATGTTTGC-3’ |
| *cFOS* | Forward | 5’-CTGTAAACCACGACCTCG-3’ |
|  | Reverse | 5’-GGAAACCAAACAGTAAACAG-3’ |
| *FOSB/ΔFOSB* | Forward | 5’-GCTAAATGCAGGAACCG-3’ |
|  | Reverse | 5’-GGTTTGTGGGCCACCAGC-3’ |
| *ID3* | Forward | 5’-GAACTTGTCATCTCCAACG-3’ |
|  | Reverse | 5’-CCAGCACCTGCGTTCTG-3’ |
| *NSRP1* | Forward | 5’-GGAAAGACAGAAAGCCCAAG-3’ |
|  | Reverse | 5’-CTTTTCCATTTATCGTTCTC-3’ |
| *SGK1* | Forward | 5’-CGATCCAAGAACCTTCTG-3’ |
|  | Reverse | 5’-CAAGATGGACTGAACTTCAG-3’ |
| *RPLP0* | Forward | 5’-CCAGCTCTGGAGAAACTG-3’ |
|  | Reverse | 5’-CTTCACATGGGGCAATGG-3’ |

**
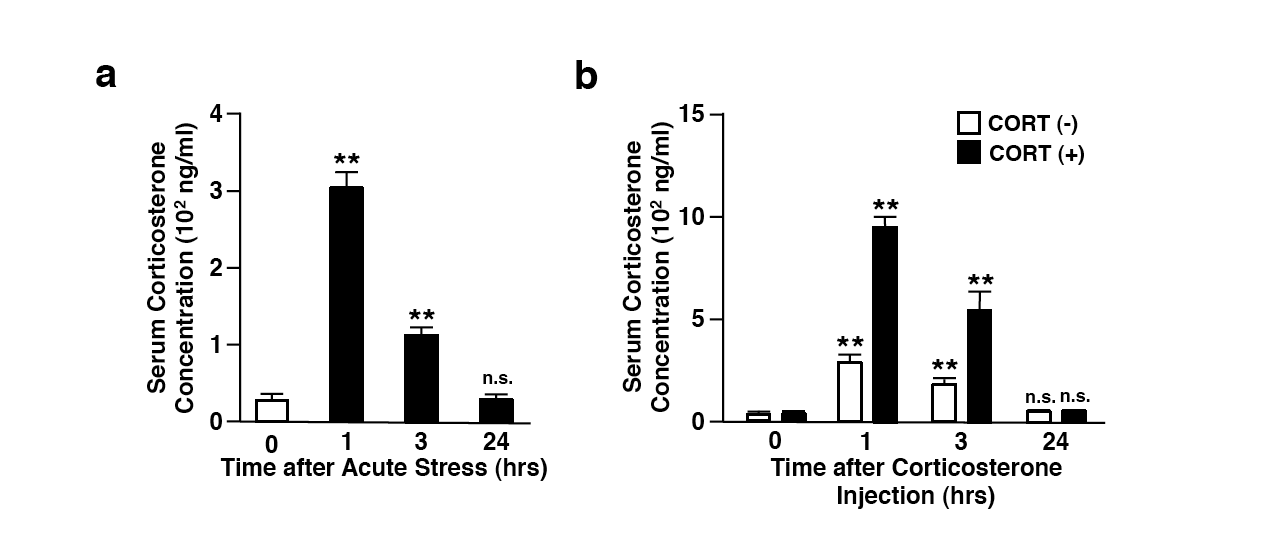
Supplemental Figure 1.** Changes of the serum corticosterone levels after acute stress or corticosterone injection in mice.

Mice were immobilized in a 50 ml falcon tube for 1 hr (acute stress) (**a**), or injected intraperitoneally with corticosterone (CORT) (20 mg/kg) (**b**), sacrificed at 0, 1, 3 and 24 hrs after the treatments, and their trunk blood was corrected for corticosterone measurement. Bars represent mean ± S.E. values of serum corticosterone levels. **: p<0.01; n.s.: not significant, compared to the data obtained at time 0.

**Supplemental Figure 2.** Acute stress differentially regulates mRNA expression of stress-related genes in mouse PFC and HIPPO.

Mice were immobilized in a 50 ml falcon tube for 1 hr, and were sacrificed at 0, 1, 3 and 24 hrs after the treatment. mRNA expression of the indicated genes was measured in PFC (**a**) and HIPPO (**b**) with the SYBR Green real-time PCR using their specific primers. Bars represented mean ± S.E. values of fold mRNA expression of indicated genes in PFC (**a**) and HIPPO (**b**). *: p<0.05, **: p<0.01, compared to the conditions indicated.
